# Relationship between trabecular bone score, bone mineral density and vertebral fractures in patients with axial spondyloarthritis

**DOI:** 10.1186/s12891-023-06431-9

**Published:** 2023-04-22

**Authors:** Elia Valls-Pascual, Ana Victoria Orenes-Vera, Ana Sendra-García, Àngels Martínez-Ferrer, Lydia Montolío-Chiva, Ignacio Vázquez-Gómez, Eduardo Flores-Fernández, Desamparados Ybáñez-García, María Vega-Martínez, Luis García-Ferrer, Magdalena Graells-Ferrer, Juan José Alegre-Sancho

**Affiliations:** 1 Rheumatology Department, Doctor Peset University Hospital, Valencia, Spain; 2 Rheumatology Department, Arnau de Vilanova University Hospital, Valencia, Spain; 3 Pharmacy Department, Doctor Peset University Hospital, Valencia, Spain; 4 Rheumatology Department, University General Hospital of Castellón, Castellón de La Plana, Spain; 5 Radiology Department, Doctor Peset University Hospital, Valencia, Spain

**Keywords:** Trabecular bone score, Bone mineral density, Axial spondyloarthritis, Syndesmophytes, Vertebral fracture

## Abstract

**Background:**

In patients with axial spondyloarthritis, vertebral fracture risk is elevated and not always correlated with bone mineral density (BMD). Trabecular bone score (TBS) may offer some advantages in the assessment of vertebral fracture risk in these patients. The primary objective of this study was to compare TBS and BMD between axial spondyloarthritis patients depending on their vertebral fracture status. Secondary objectives were to estimate the prevalence of morphometric vertebral fractures, and to explore factors associated with fracture, as well as the interference of syndesmophytes on BMD and TBS.

**Methods:**

A cross-sectional study was conducted. Data were collected on demographic and clinical characteristics, lab results, imaging findings and treatment. Statistical analysis was performed using SPSS v.13 statistical software.

**Results:**

Eighty-four patients (60 men and 24 women; mean age of 59 years) were included. Nearly half (47.6%) of them had lumbar syndesmophytes. The rate of morphometric fracture was 11.9%. TBS showed a higher area under the curve (0.89) than total hip, femoral neck and lumbar BMD (0.80, 0.78, and 0.70 respectively) for classifying patients regarding their fracture status. Nonetheless, the differences did not reach statistical significance.

Syndesmophytes affected lumbar spine BMD (*p* < 0.001), but not hip BMD or TBS. Fractures were associated with TBS, total hip BMD, erythrocyte sedimentation rate and C-reactive protein levels.

**Conclusions:**

We identified decreased TBS and total hip BMD, as well as increased erythrocyte sedimentation rate and C-reactive protein levels as factors associated with morphometric vertebral fractures. Unlike lumbar spine BMD, TBS is not affected by the presence of syndesmophytes.

## Background

Axial spondyloarthritis (axSpA) is a chronic disease characterized by the involvement of the axial skeleton. In later stages of the disease, a combination of sustained inflammation and structural damage may lead to decreased bone mass and impaired bone microarchitecture, affecting bone strength. An elevated risk of vertebral fracture has been described among patients with axSpA [1], with estimated prevalence rates of morphometric vertebral fracture as high as 31% [2]. Such high rates have been related to inflammatory activity and structural damage, which may lead to a general loss of bone mass and increased stiffness of the spine [3, 4].

Bone mineral density (BMD) assessment by dual-energy X-ray absorptiometry (DXA) is considered the gold standard for the diagnosis of osteoporosis. Patients with non-radiographic axSpA, showed lower lumbar BMD than age- and sex-matched controls [5]. However, this is not the case in patients with advanced axSpA, in whom the presence of syndesmophytes may interfere with BMD, as previously demonstrated [6, 7].

In addition to BMD, there are other DXA-derived skeletal parameters which can provide information on bone quality. Bone strain index is a parameter of bone deformation that can be applied both to lumbar and femoral DXA scans and has been proven useful in identifying patients at risk of fracture [8]. Hip structure analysis, hip axis length and neck shaft angle are methods of estimating hip geometry and biomechanical parameters using data obtained from DXA scans of the hip. They have shown to be predictors of hip fracture [9–11].

Inflammation has been suggested to have a direct negative effect on vertebral trabecular bone [12]. Changes in the trabecular structure can be assessed by quantitative computed tomography (QCT) and high-resolution peripheral QCT (HR-pQCT), both being useful for vertebral fracture prediction [13, 14]. Nonetheless, these techniques involve high radiation exposure and may not be available in routine clinical practice.

The trabecular bone score (TBS) is a textural index that evaluates pixel gray-level variations in the lumbar spine DXA image, using specific software. It provides an indirect measure of trabecular microstructure [15]. A correlation between TBS and QCT has been demonstrated [16] and, based on the results of previous studies, it appears that TBS is not affected by the presence of syndesmophytes [12, 17]. Notably, patients with axSpA and vertebral fractures were found to have significantly lower TBS values than those without fractures [18]. Given this, we considered elucidating whether TBS may offer some advantage over BMD for estimating vertebral fracture risk in patients with axSpA.

The objectives of this study were to compare TBS and BMD between axSpA patients depending on their vertebral fracture status, to estimate the prevalence of morphometric vertebral fractures, and to explore factors associated with fracture, as well as the interference of syndesmophytes on BMD and TBS in a cohort of patients with axSpA.

## Methods

### Study design and population

A cross-sectional study was performed. The available population was all patients with axSpA followed up at the Department of Rheumatology of the Doctor Peset University Hospital (Valencia, Spain). Those who met the following criteria were consecutively selected: diagnosis of axSpA according to Assessment of SpondyloArthritis International Society (ASAS) criteria, 18 years of age or older, body mass index (BMI) < 37 kg/m^2^ (the highest permitted according to the technical specifications of our DXA device), and eligibility for DXA (absence of both bilateral hip replacement and instrumented lumbar spinal fusion).

### Calculation of sample size

We used the calculator available on the website of the Hospital del Mar Medical Research Institute (GRANMO) [19]. A recent study [18] found that 16% of patients with axSpA and vertebral fractures had a BMD in the range considered indicative of osteoporosis, while about 29% had a low TBS. Considering an alpha of 0.05 and beta of 0.2 in two-tailed tests, a sample of 76 individuals would be required to detect differences of 0.3 units or more. We assumed that the proportion in the reference group was 0.16 and estimated a dropout rate of 5%.

### Variables

The main outcome variable was the presence of morphometric vertebral fractures. This was determined by the evaluation of a plain lateral radiograph of the dorsolumbar spine, obtained using an AGFA DR 400 system. We defined vertebral fracture as a vertebral height loss of ≥ 25% (grades 2 and 3 in Genant’s semi-quantitative classification method [20]), as this appears to increase specificity compared to a lesser degree of deformity [21]. All images were evaluated by the same reader, a rheumatologist with 15 years of experience, trained by radiologists and specially focused on spine radiologic findings.

BMD and TBS were measured using a Lunar Prodigy Pro bone densitometer (GE Healthcare) and TBS iNsight® software, version 2.2, respectively. Calibration of the densitometer was daily performed using a quality assurance (QA) block, showing a coefficient of variation of 0.25%. Estimates of BMD were obtained for the lumbar spine (anterior–posterior position), the total hip and the femoral neck. BMD was expressed as the absolute value and standard deviation (SD) of the mean for the young adult population of the same sex and geographical area (T-score) or the mean for the population of the same age range in the case of premenopausal women and men under 50 years of age (Z-score).

According to the World Health Organization criteria [22], osteopenia was defined as a T score between -1 and -2.5, and osteoporosis as a T score of -2.5 or less. Patients were divided into three groups according to this criterion. In pre-menopausal women and men under 50, patients were divided based on the Z-score. A Z-score of -2.0 or lower was defined as “below the expected range for age”, and a Z-score above -2.0 as “within the expected range for age” [23]. For patients with lumbar vertebral fractures, we examined the results excluding the fractured vertebrae.

We classified TBS using the ranges described in the meta-analysis published by McCloskey et al. [24]: normal for TBS ≥ 1.3; moderate for 1.3 > TBS > 1.23; and low for TBS ≤ 1.23. For the inferential statistical analysis, patients were divided into two groups: patients with a TBS ≤ 1.23 (low TBS) versus > 1.23 (not low TBS). Absolute values were also analysed.

We collected data on the following descriptive variables: age, sex, BMI, disease duration, scores on the Bath Ankylosing Spondylitis Disease Activity Index (BASDAI), Bath Ankylosing Spondylitis Disease Functional Index (BASFI), erythrocyte sedimentation rate (ESR), levels of C-reactive protein (CRP), 25-hydroxy vitamin D (25-OH-D), calcium, parathyroid hormone (PTH), alkaline phosphatase (ALP), and β-CrossLaps (β-CTX) in blood, and 24-h urinary calcium, presence of lumbar syndesmophytes and/or osteophytes, and use and type of treatment for axSpa and osteoporosis.

The presence of syndesmophytes and osteophytes was evaluated on a plain radiograph of the dorsolumbar spine (anteroposterior and lateral views) obtained with the same AGFA DR 400 system as the one used to detect fractures. All images were evaluated by the same reader.

### Statistical analysis

Percentage and 95% confidence interval (95% CIs) were calculated for categorical variables and mean and standard deviation (SD) or median and interquartile range (IQR) for continuous variables, depending on the distribution of the data. Percentages were compared using chi-square tests with a continuity correction when appropriate. Means were compared using Student’s t tests or analysis of variance when data were normally distributed, and otherwise with Kruskal–Wallis/Mann–Whitney U tests. *P* values < 0.05 were considered significant. Logistic regression analysis was used to study the factors associated with the presence of vertebral fractures.

The accuracy of TBS and BMD in classifying patients regarding their fracture status was assessed by determining the area under the receiving operator characteristics (ROC) curve. Sensitivity and specificity as well as the likelihood ratios for positive and negative test results with TBS and BMD were calculated. Statistical analysis was performed using SPSS v.13 statistical software.

### Ethical considerations

The Ethics Committee of the Doctor Peset University Hospital approved the study (date of authorisation: 12/09/2018; study code: 18/065).

The study was classified as a non-post-authorisation study (non-PAS) by the Spanish Agency of Medicines and Medical Products (AEMPS).

All eligible patients were informed verbally and in writing about the study. Prior to inclusion, all participants provided written informed consent in accordance with the Declaration of Helsinki and received a copy of the form they had signed.

## Results

A total of 84 patients were included, 60 men and 24 women, with a mean age of 59 years (± SD 13). Nearly half of them (47.6%; 95% CI [37.1, 58.3]) had lumbar syndesmophytes. Ten patients had vertebral fractures, corresponding to a prevalence of 11.9% (95% CI [6.2, 20.2]). Just over half of the patients (51.2%; 95% CI [40.6, 61.8]) were treated with nonsteroidal anti-inflammatory drugs and just under half of them (48.8%; 95% CI [38.3, 59.5]) with biologics. Disease duration was greater than 10 years in almost two-thirds of patients (65.5%; 95% CI [54.9, 75]). These and other parameters describing the baseline characteristics of the sample are reported in Table 1. Total hip and femoral neck BMD and TBS data were not available for three patients, due to technical problems. Lumbar spine BMD was not available for one patient due to L1 to L4 vertebral fractures.

**Table 1:** Baseline characteristics of patients included in the study

Baseline characteristics of the sample	
Sex, n (%;95% CI)	♂ 60 (71.4; 61.1, 80.3) ♀ 24 (28.6; 19.7, 38.9)
Age in years, mean (± SD)	59 (± 13)
Body mass index in kg/m^2^, mean (± SD)	28.9 (± 4.7)
Presence of syndesmophytes, n (%;95% CI)	40 (47.6; 37.1, 58.3)
Presence of osteophytes, n (%;95% CI)	18 (21.4; 13.6, 31.2)
Treatment with NSAIDs, n (%;95% CI)	43 (51.2; 40.6, 61.8)
Treatment with biologics, n (%;95% CI)	41 (48.8; 38.3, 59.5)
Disease duration in range of years, n (%;95% CI)	
> 10 years	55 (65.5; 54.9, 75)
5–10 years	17 (20.2; 12.7, 29.8)
< 5 years	12 (14.3; 7.9, 23)
BASDAI, mean (± SD) (*n* = 52)	3.7 (± 2.2)
BASFI, mean (± SD) (*n* = 52)	4.3 (± 2.3)
ASDAS, mean (± SD) (*n* = 38)	2.7 (± 0.9)
CRP mg/L, median (IQR)	5 (53)
ESR mm/h, median (IQR)	9 (57)
25-OH-D ng/dL, median (IQR) (*n* = 78)	24 (66)
Blood calcium mg/dL, mean (± SD) (*n* = 76)	9.4 (± 0.4)
24-h urinary calcium, median (IQR) (*n* = 33)	143 (421)
PTH pg/mL, median (IQR) (*n* = 40)	47 (114)
ALP UI/L, median (IQR) (*n* = 70)	71.5 (180)
β-CTX pg/mL, median (IQR) (*n* = 45)	374 (901)
Lumbar spine T-score	
Osteoporosis, n (%;95% CI)^a^	6 (7.2; 3, 14.4)
Osteopenia, n (%;95% CI)^a^	10 (12;6.3, 20.4)
Hip T-score	
Osteoporosis, n (%;95% CI)^a^	12 (14.8; 8.3, 23.9)
Osteopenia, n (%;95% CI)^a^	18 (22.2; 14.2, 32.2)
Lumbar spine Z-score	
Low bone mass, n (%;95% CI)^a^	2 (2.4;0.4, 7.7)
Hip Z-score	
Low bone mass, n (%;95% CI)^a^	0
TBS range (*n* = 81)	
Low, n (%;95% CI)	14 (17.3; 10.2, 26.7)
Intermediate, n (%;95% CI)	18 (22.2; 14.2, 32.2)
Presence of vertebral fracture, n (%;95% CI)	10 (11.9; 6.2, 20.2)

*n* number of patients, *CI* confidence interval, *SD* standard deviation, *IQR* interquartile range, *NSAIDs* nonsteroidal anti-inflammatory drugs, *BASDAI* Bath Ankylosing Spondylitis Disease Activity Index, *BASFI* Bath Ankylosing Spondylitis Disease Functional Index, *ASDAS* Ankylosing Spondylitis Disease Activity Score, *CRP* C-reactive protein, *ESR* erythrocyte sedimentation rate, *25-OH-D* 25-hydroxy vitamin D, *PTH* parathyroid hormone, *ALP* alkaline phosphatase, *β-CTX* β-CrossLaps (β-CTX), T-score: standard deviation of the mean for the young adult population of the same sex and geographical area, Z-score: standard deviation of the mean for the population of the same age range, TBS: trabecular bone score. ^a^Percentages were calculated based on the total study sample with available data. Mean and standard deviation have been described for variables showing normal distribution and median and interquartile range for variables showing not normal distribution

Among patients with vertebral fractures, four of them received their first osteoporosis treatment (oral bisphosphonates) at the time of fracture diagnosis. One patient was not receiving treatment with no apparent reason. The remaining five patients started their first treatment (4 started an oral bisphosphonate and 1 bazedoxifene) due to a low BMD, switching to a second drug when vertebral fracture was detected (oral bisphosphonate, strontium ranelate, denosumab in 1 case each, and zoledronic acid in 2 cases).

**Fig. 1:**
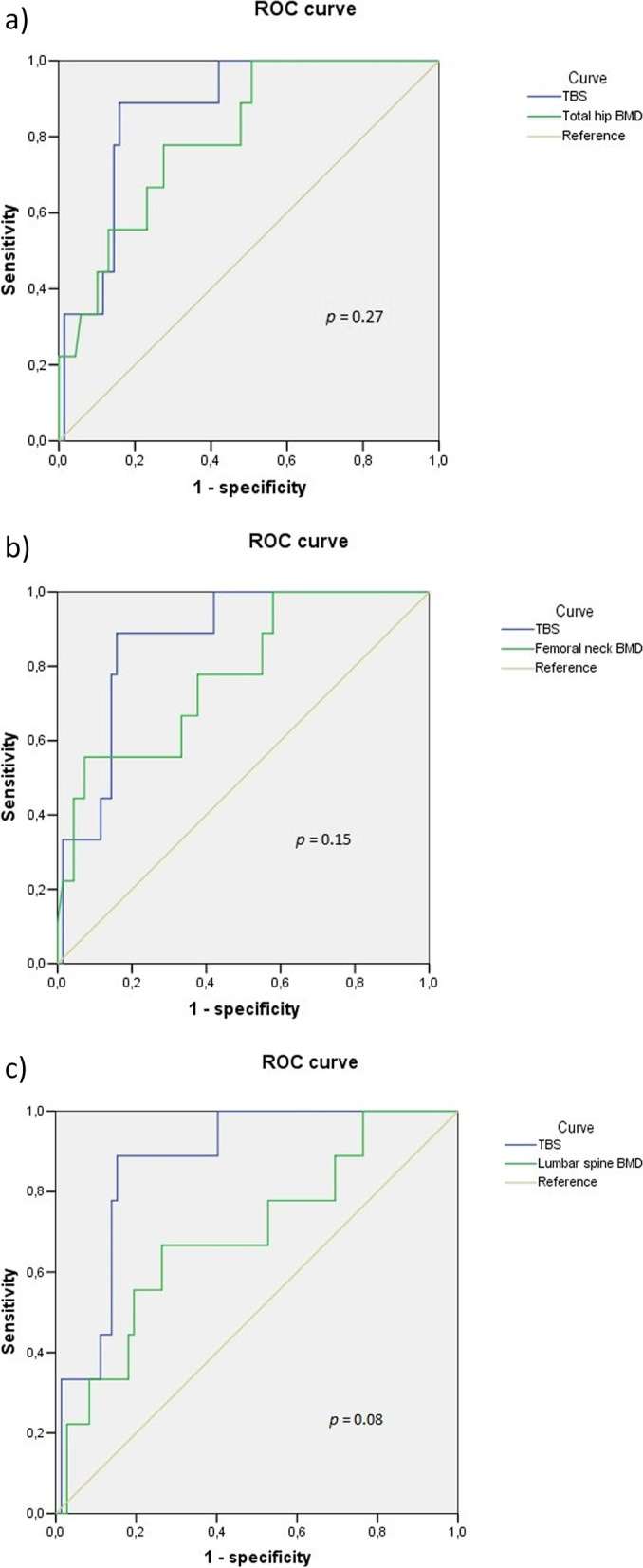
ROC curves of TBS and BMD for the discrimination of vertebral fractures. **a** Comparison between TBS and total hip BMD in the discrimination of vertebral fractures. **b** Comparison between TBS and femoral neck BMD in the discrimination of vertebral fractures. **c** Comparison between TBS and lumbar spine BMD in the discrimination of vertebral fractures. ROC curve: receiver operating characteristic curve; TBS: trabecular bone score; BMD: bone mineral density

The accuracy of TBS and BMD in classifying patients according to their fracture status is represented in the ROC curves shown in Fig. 1. The area under the curve was larger for TBS (0.89) than for total hip, femoral neck and lumbar BMD (0.80, 0.78, and 0.70 respectively). Nonetheless, the differences did not reach statistical significance.

TBS showed a sensitivity of 66.7% (95% CI [35.4, 87.9]) higher than that of BMD, both in the lumbar spine (11.1%; 95% CI [1.9, 43.5]) and the hip (33.3%; 95% CI [12.1, 64.6]). Its specificity (85.5%; 95% CI [75.3, 91.9]) was slightly lower than that of lumbar spine BMD (91.3%; 95% CI [82.3, 95.9]) but similar to that of hip BMD (86.9%; 95% CI [77.1, 92.9]]. The likelihood ratios were 4.4, 1.23, and 2.13 for positive test results and 0.51, 0.98 and 0.82 for negative test results for TBS, lumbar spine BMD and hip BMD respectively.

Patients with syndesmophytes were older (*p* = 0.04), had a higher BMI (*p* < 0.001), and were more likely to be male (*p* < 0.001) than patients without syndesmophytes. Comparisons of these and other baseline characteristics between patients with and without syndesmophytes are shown in Table 2.

**Table 2:** Differences in baseline characteristics of patients included in the study regarding syndesmophyte status

Variable	Syndesmophytes	No syndesmophytes	*p* Value
Sex, n ♂ / n ♀	38 / 2	22 / 22	< 0.001
Age in years, mean (± SD)	62 (± 12)	56.2 (± 13.6)	0.004
Body mass index in kg/m^2^, mean (± SD)	30.7 (± 4.5)	27.3 (± 4.4)	0.001
Treatment with NSAIDs / biologics, n (%;95% CI)	22 (55; 39.5, 69.8) / 18 (45; 30.2, 60.5)	21 (47.7; 33.3, 62.4) / 23 (52.3; 37.6, 66.6)	0.505
Disease duration in range of years, n (%;95% CI)			
> 10 years 5–10 years < 5 years	27 (67.5; 51.9, 80.6) 9 (22.5; 11.6; 37.3) 4 ( 10; 3.26, 22.4)	28 (63.6; 48.7, 76.8) 8 (18.2; 8.8, 31.6) 8 (18.2; 8.8, 31.6)	0.543
BASDAI, mean (± SD) (*n* = 22/30)	3.8 (± 2.3)	3.6 (± 2.2)	0.651
BASFI, mean (± SD) (*n* = 23/29)	4.7(± 2)	3.9 (± 2.6)	0.225
ASDAS, mean (± SD) (*n* = 18/20)	2.7 (± 0.9)	2.7 (± 0.8)	0.842
CRP mg/L, median (IQR)	5 (48)	5 (53)	0.731
ESR mm/h, median (IQR)	10 (57)	8 (50)	0.435
25-OH-D ng/dL, median (IQR) (*n* = 38/40)	23 (66)	24.5 (55)	0.339
Blood calcium mg/dL, mean (± SD) (*n* = 38/38)	9.37 (± 0.3)	9.33 (± 0.39)	0.669
24-h urinary calcium, median (IQR) (*n* = 18/15)	125 (317)	145 (381)	0.448
PTH pg/mL, median (IQR) (*n* = 23/17)	47 (107)	49 (65)	0.891
ALP UI/L, median (IQR) (*n* = 31/39)	73 (180)	71 (77)	0.242
β-CTX pg/mL, median (IQR) (*n* = 23/22)	352 (901)	396.5 (522)	0.394
TBS values median (IQR) (*n* = 39/42)	1.31 (0.58)	1.33 (0.8)	0.187
Lumbar spine BMD values mean (± SD)	1.37 (± 0.26)	1.14 (± 0.22)	< 0.001
Total hip BMD values mean (± SD) (*n* = 38/43)	0.99 (± 0.17)	0.96 (± 0.16)	0.463
Femoral neck BMD values mean (± SD) (*n* = 38/43)	0.88 (± 0.14)	0.87 (± 0.14)	0.820

*n* number of patients, *SD* standard deviation, *IQR* interquartile range, *NSAIDs* nonsteroidal anti-inflammatory drugs, *CI* confidence interval, *BASDAI* Bath Ankylosing Spondylitis Disease Activity Index, *BASFI* Bath Ankylosing Spondylitis Disease Functional Index, *ASDAS* Ankylosing Spondylitis Disease Activity Score, *CRP* C-reactive protein, *ESR* erythrocyte sedimentation rate, *25-OH-D* 25-hydroxy vitamin D, *PTH* parathyroid hormone, *ALP* alkaline phosphatase, *β-CTX* β-CrossLaps (β-CTX), *TBS* trabecular bone score, *BMD* bone mineral density. Mean and standard deviation have been described for variables showing normal distribution and median and interquartile range for variables showing not normal distribution

The presence of syndesmophytes significantly affected lumbar BMD (*p* < 0.001), but not TBS. In contrast, we found no interference of osteophytes on BMD or TBS.

In the univariate analysis, vertebral fractures were associated with sex, total hip and femoral neck BMD, TBS, and ESR and CRP levels. The sample was too small to assess the potential influence of disease duration or the presence of osteophytes. When adjusted for age and sex, vertebral fractures were found to be related to TBS, total hip BMD, erythrocyte sedimentation rate and C-reactive protein levels. Odds ratios, confidence intervals and significance levels are reported in Table 3.

**Table 3:** Study of factors associated with the presence of vertebral fracture

**Variable**	**OR**	**95% CI**	***p*** ** Value**
Sex	4.67	[1.18, 18.40]	0.03
Total hip BMD	0.43	[0.24, 0.75]	0.003
Femoral neck BMD	0.41	[0.21, 0.78]	0.007
TBS	0.16	[0.05, 0.49]	0.001
CRP	1.06	[1.00, 1.13]	0.04
ESR	1.08	[1.03, 1.14]	0.003
Age	1.05	[0.99, 1.12]	0.08
Lumbar spine BMD	0.71	[0.51, 1.00]	0.05
ALP	1.02	[1.00, 1.04]	0.08
24-h urinary calcium	0.98	[0.97, 1.00]	0.07
**Variables adjusted by age and sex**	**OR**	**95% CI**	***p*** ** Value**
Total hip BMD	0.48	[0.26, 0.89]	0.02
TBS	0.15	[0.04, 0.52]	0.003
CRP	1.08	[1.01, 1.15]	0.02
ESR	1.07	[1.02, 1.14]	0.01

*OR* odds ratio, *CI* confidence interval, *BMD* bone mineral density, *TBS* Trabecular Bone Score, *CRP* C-reactive protein, *ESR* erythrocyte sedimentation rate, *ALP* alkaline phosphatase

## Discussion

The prevalence of morphometric vertebral fractures in our sample was 11.9%, very similar to that described in the study of Geusens et al. [25]. A higher prevalence of radiologic vertebral fractures has been described among patients with axSpA in a study published in 2014 [2]. It should be noted that in this study the sample was small, vertebral fracture was defined as a vertebral height loss of ≥ 20% rather than ≥ 25%, and the patients included were those diagnosed with ankylosing spondylitis according to the New York criteria, probably reflecting higher spinal structural damage which is known to be related to the pathogenesis of vertebral fracture in spondylitis patients.

In our study, in line with previous research [7, 15], the presence of syndesmophytes affected lumbar spine BMD but not TBS. The difference in lumbar BMD between patients with and without syndesmophytes could be partly explained by the lower percentage of women in the group with syndesmophytes than in the group without. To further assess the potential interference of spinal bone formation on these parameters, we also considered osteophytes, but did not detect any effect. This partially differs from the results of previous studies [26, 27]. A potential explanation for the lack of effect of osteophytes on lumbar spine BMD is that we only considered the presence or absence of osteophytes, rather than the extent of new bone formation, whereas this has been assessed in the aforementioned studies.

Bone microstructural changes assessed using various methods, including TBS, have shown to be predictive of vertebral fractures, independent of BMD, in both women and men [14, 16, 24, 28–30]. In our study, TBS, total hip BMD, ESR and CRP levels have been identified as factors independently associated with the presence of vertebral fractures. Due to the cross-sectional design of this study, the predictive value of TBS could not be assessed.

To the best of our knowledge, our work is the first to compare TBS and BMD between axSpA patients depending on their vertebral fracture status in a Spanish cohort. Two previous studies addressed this question, based on cohorts from South Korea and Canada [18, 31]. The results of these studies suggest that TBS has a potential role in predicting vertebral fracture in axSpA patients. In our study, TBS was found to have higher sensitivity and comparable specificity than BMD in classifying patients for fracture status. The area under the curve was higher for TBS, although the difference with BMD did not reach statistical significance, probably due to the low sample size of our cohort.

All these results suggest that it would be worth assessing bone microarchitecture with TBS software for the estimation of the risk of vertebral fracture in patients with axSpA.

### Limitations

The main limitation of our study is the scarce number of fractures found, probably determined by the small sample size. This could have influenced some of our results. First, it could have affected the accuracy of TBS and BMD in discriminating prevalent vertebral fractures. In second place, it could have influenced the relationship between clinical and laboratory factors and the presence of vertebral fractures.

The disproportion between men and women represents another limitation of our study, as the bone quality between the two differs significantly, which affects the susceptibility of vertebral fractures.

Regarding laboratory variables, we should note that we considered the most recent values recorded in the medical history, rather than an average of the values over time, which could better reflect each parameter’s status.

In addition, it should be mentioned that we were unable to analyse the interference of osteoporosis treatment in the presence of fractures due to the low sample size and the design of our study.

Further, due to the cross-sectional design of the study, an analysis of TBS for fracture prediction as an incident event could not be performed.

## Conclusions

In our study, decreased TBS and total hip BMD, as well as increased ESR and CRP levels have been identified as factors independently associated with the presence of vertebral fractures.

Unlike lumbar spine BMD, TBS is not affected by the presence of syndesmophytes.

## Data Availability

The datasets used and/or analysed during the current study are available from the corresponding author on reasonable request.

## Abbreviations

axSpA: Axial spondyloarthritis
BMD: Bone mineral density
DXA: Dual-energy X-ray absorptiometry
CT: Computed tomography
TBS: Trabecular bone score
ASAS: Assessment of SpondyloArthritis International Society
BMI: Body mass index
SD: Standard deviation
T-score: Mean BMD for the young adult population of the same sex and geographical area
Z-score: Mean BMD for the population of the same age range in the case of premenopausal women and men under 50 years of age
BASDAI: Bath Ankylosing Spondylitis Disease Activity Index
BASFI: Bath Ankylosing Spondylitis Disease Functional Index
ESR: Erythrocyte sedimentation rate
CRP: C-reactive protein
25-OH-D: 25-Hydroxy vitamin D
PTH: Parathyroid hormone
ALP: Alkaline phosphatase
β-CTX: β-CrossLaps
95% CI: 95% Confidence interval
ROC curve: Receiver operating characteristic curve
